# The value of cardiac CT in the diagnosis of unroofed coronary sinus syndrome

**DOI:** 10.1186/s12872-022-02966-2

**Published:** 2022-12-02

**Authors:** Junqing Ma, Yongze Zheng, Sunan Xu, Hewei Teng, Lei Lv, Yanpei Li, Yongfeng Liang, Yang Zhang

**Affiliations:** Department of Radiology, Qilu Hospital of Shandong University, Jinan, China

**Keywords:** Unroofed coronary sinus syndrome, Cardiac CT, Postprocessing technique, Transthoracic echocardiography

## Abstract

**Background:**

Unroofed coronary sinus syndrome (UCSS) is a rare cardiovascular malformation with nonspecific clinical manifestations that easily causes misdiagnosis and missed diagnosis. The aim of this study is to present the different features of UCSS by various CCT (cardiac CT) postprocessing techniques and evaluate the diagnostic advantages of CCT.

**Methods:**

9 UCSS patients who were diagnosed by imaging and undergone both CCT and transthoracic echocardiography (TTE) were included in this study, and their CCT images were reviewed. The UCSS images were classified by multiplanar reformations, maximum intensity projection, volume rendering and cinematic rendering. The size of CS roof defect was also measured.

**Results:**

Only 4 of 9 CCT confirmed UCSS patients were detected by TTE (4/9, 44.4%), the sensitivity of TTE was lower compared to CCT by Fisher’s exact test (*P* < 0.05). UCSS was classified according to the Kirklin and Barratt Boyes’s method, including 1 case was classified as type I, 4 cases as type II, 1 case as type III, 2 cases as type IV, 1 case as type V (variant type), and TTE was undiagnosed in all type III-V patients. Additionally, CCT showed 12 extra malformations in these patients, only 5 of them were found by TTE (5/12, 41.7%), and TTE missed all extracardiac malformations. The mean size of CS roof defect was 3.04 ± 1.57 cm.

**Conclusions:**

CCT with various postprocessing technologies has excellent value in diagnosing and differentiating subtypes of UCSS, measuring size of coronary sinus defect, describing accompanying cardiovascular abnormalities.

## Introduction

The coronary sinus (CS) has no traffic with the left atrium (LA), and it courses in the coronary sulcus between the left atrium and the left ventricle (LV), collecting the majority of the myocardiac blood. The main branches of the CS are the great cardiac vein (GCV), the middle cardiac vein (MCV), the small cardiac vein (SCV), oblique vein of left atrium (OVLA), the left marginal vein (LMV), and the left posterior vein (Lpv). UCSS, a rare type of atrial septal defect (ASD), is formed anatomically by complete or partial absence of the CS roof, which causes CS connect to LA. In some patients, UCSS is accompanied with the persistent left superior vena cava (PLSVC), and sometimes the left and right superior vena cava can be connected through innominate vein (Inn.V.) [1]. If a right-to-left shunt occurs in UCSS, it may increase the risk of cerebral embolism and brain abscess, thus it is important to diagnose the disease early and accurately [2]. Currently, transthoracic echocardiography (TTE) is the most common method for the diagnosis of cardiac malformation. However, it is difficult to show the defect of CS located posterior to the heart by TTE due to the limited acoustic window. TTE is not ideal for the diagnosis of extracardiac malformation such as PLSVC and anomalous pulmonary venous connection [3, 4]. Compared to TTE, cardiac computer tomography (CCT) with superior spatial resolution shows advantages of evaluating the coronary vein system, and exhibiting the abnormalities of intracardiac structure and extracardiac vascularity [5–7]. The aim of this study is to present the different features of UCSS by various CCT postprocessing techniques and evaluate the diagnostic advantages of CCT.

## Materials and methods

With the approval of the hospital ethics committee, the database of our hospital from May 1st, 2016 to October 31st, 2021 was retrospectively screened. Among 5859 patients with congenital heart disease, a total of 14 UCSS patients diagnosed by imaging following the diagnostic criteria in previous publications [1, 6, 8–10]. CS roof wall defect or the anomalous vein connecting the LA to the CS observed on TTE or CCT was served as evidence for diagnosis. 9 patients who had undergone both CCT and transthoracic echocardiography (TTE) were included in this study. The case-related ultrasonic examination data and clinical data were from the patients' medical records.

### CCT scan

Cardiac ECG-gated CT was engaged on a dual-source scanner (128-MDCT, SOMATOM Definition Flash, Siemens Healthcare) in this study. Scan range was from 10–15 mm below the tracheal bifurcation to the diaphragmatic surface of the heart. Tube voltage and tube current were regulated by Care Dose 4D and Care KV technology. The pitch was adjusted according to the heart rate, with rack speed of 0.25 s, layer thickness of 0.75 mm, and reconstruction interval 0.50 mm. The image series of optimal diastolic and systolic phase was reconstructed automatically or selectively. Nonionic contrast agent (Iopromide 370, Bracco) in a dosage of 0.7 ml/kg body weight at 5 ml/s was injected by double-barrel high-pressure syringe following with 40 ml sanitary saline at the same rate. The duration of contrast agent injection was 6-13 s. Scanning was triggered 6 s after the CT value of ascending aorta reached 100 hounsfield (HU) by group note tracking procedures technology.

### Image analysis

Image postprocessing was carried out on Syngo.via workstation with postprocessing techniques: multiplanar reformations (MPR), maximum intensity projection (MIP), volume rendering (VR), cinematic rendering (CR). The short axis, long axis, oblique coronal and sagittal images of the coronary sinus and PLSVC were reconstructed. The size of CS roof defect was measured on coronal MPR images. UCSS was classified according to the Kirklin and Barratt Boyes’s method [1]: Type I: complete absence of parietal wall of coronary sinus with PLSVC; Type II: complete absence of parietal wall of coronary sinus without PLSVC; Type III: perforation of the middle segment of the parietal wall of coronary sinus; Type IV: perforation of the parietal wall of the end segment of coronary sinus. And the patient with abnormal venous connection between the CS and LA was regarded as the variant type (type V). All the original CCT images and the postprocessed images were jointly read and acknowledged by two radiologists, who did not know the actual diagnosis and surgical results.

### Statistical analysis

The data was processed by GraphPad Prism 8 software. The sensitivity of CCT and TTE for the diagnosis of UCSS was compared by Fisher's exact test, *P* < 0.05 was considered statistically significant.

## Results

### Clinical characters of UCSS

There were 9 patients with UCSS, aged from 30 to 56 years, with an average of 48.1 ± 8.0 years, included 3 males and 6 females, the clinical characteristics were summarized in Table 1, in which cyanosis, dyspnea, palpitations, cardiac murmur and arrhythmias were common.

**Table 1:** Summary of clinical characteristics of UCSS

Patient no	Sex	Age (y)	Clinical Symptoms	Heart Sounds And Murmurs	Arrhythmia
1	M	50	Cyanosis/Dyspnea	P2 loud	f-AVB/IRBBB
2	F	46	Cough	PASM	IRBBB
3	F	55	Cyanosis/Dyspnea	P2 loud/PASM/TSM/MSM	AF/LAH/CRBBB
4	F	56	None	P2 loud	IRBBB
5	F	56	None	MSM	None
6	M	44	Palpitations/Dyspnea	TSM	None
7	F	43	Palpitations/Dyspnea	P2 loud/P2 splitting/PASM/TSM	None
8	F	30	Palpitations/Dyspnea	PASM	VPB
9	M	53	Palpitations/Dyspnea	None	None

*F* female, *M* male, *P2* pulmonary second heart sound, *PASM* pulmonary area systolic murmur, *TSM* tricuspid systolic murmur, *MSM* mitral systolic murmur, *f-AVB* first degree atrioventricular block, *IRBBB* incomplete right bundle branch block, *AF* atrial fibrillation, *LAH* left anterior hemiblock, *CRBBB* complete right bundle branch block *VPB* ventricular premature beat

### Image characteristics

**Fig. 1:**
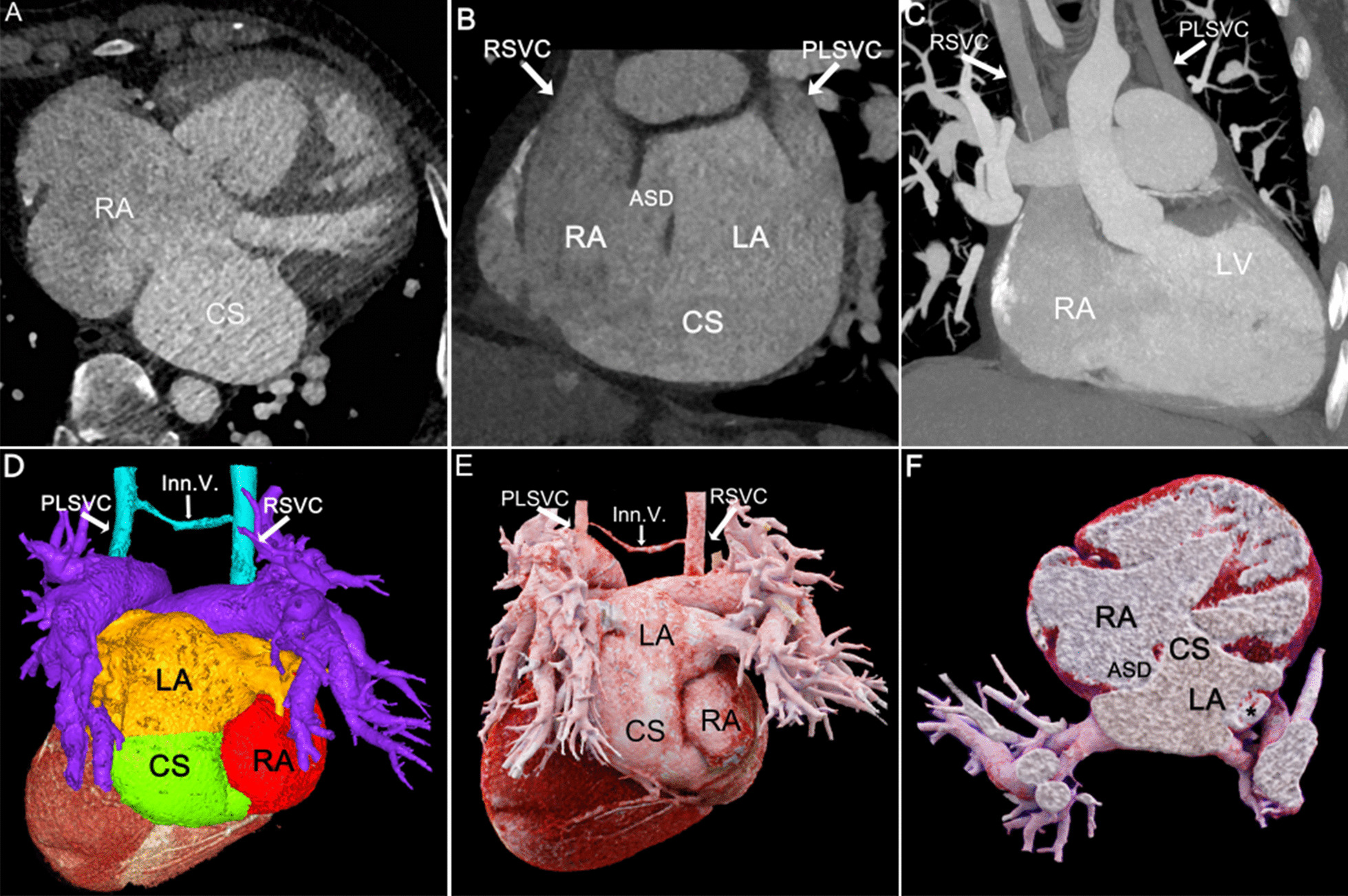
Patient No.1, type I: **A** MPR axial view showed enlargement of the CS. **B**, **C** MPR and MIP showed ASD and PLSVC. The CS communicated LA to RA. **D**, **E** VR color rendering and CR showed complete absence of the parietal wall of the CS. PLSVC was connected to RSVC via Inn.V. **F** CR cross-sectional view visualized the spatial relationship between the ASD and the CS defect. RA right atrium, and the section of PLSVC(_*_) flowing into the left atrium also displayed. CS coronary sinus, LA left atrium, RSVC right superior vena cava, PLSVC persistent left superior vena cava, ASD atrial septal defect, Inn.V. innominate vein

**Fig. 2:**
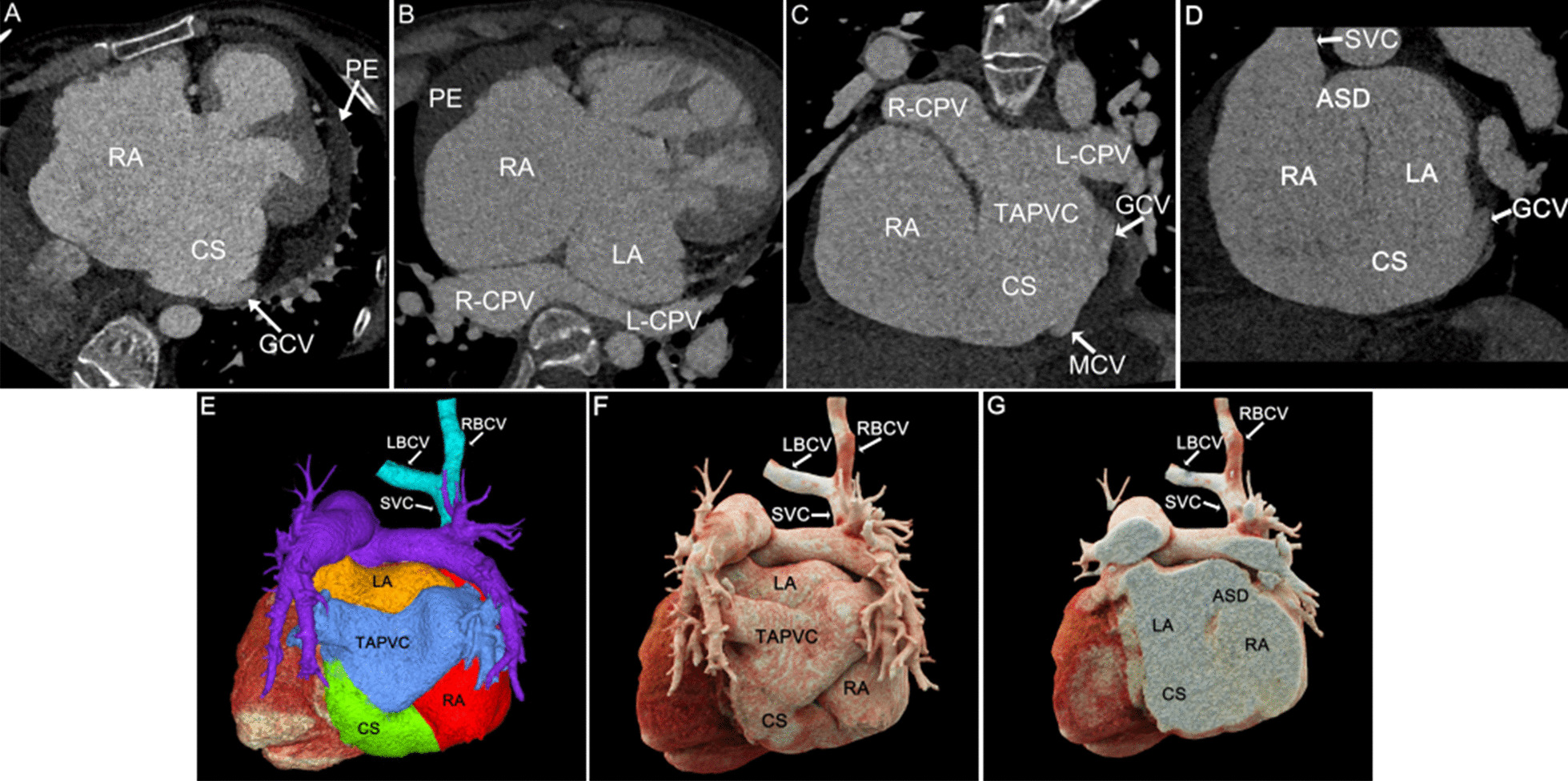
Patient No.3, type II: **A** MPR axial view showed enlargement of the CS. **B**, **C** MPR axial and coronal views showed L-CPV and R-CPV ectopically drained into the enlarged CS, and formed cor triatrium. **D**: MPR oblique view was a representation of the ASD and the defected CS, **E**, **F** VR and CR directly showed the UCSS and TAPVC, and SVC is displayed normally but no PLSVC exists. **G**: CR cross-sectional view showed the UCSS and ASD. RA right atrium, CS coronary sinus, GCV great cardiac vein, PE pericardial effusion, LA left atrium, L-CPV left common pulmonary vein, R-CPV right common pulmonary vein, TAPVC total anomalous pulmonary vena cava, SVC superior vena cava, LBCV left brachiocephalic vein, RBCV right brachiocephalic vein, MCV middle cardiac vein, GCV great cardiac vein, ASD atrial septal

**Fig. 3:**
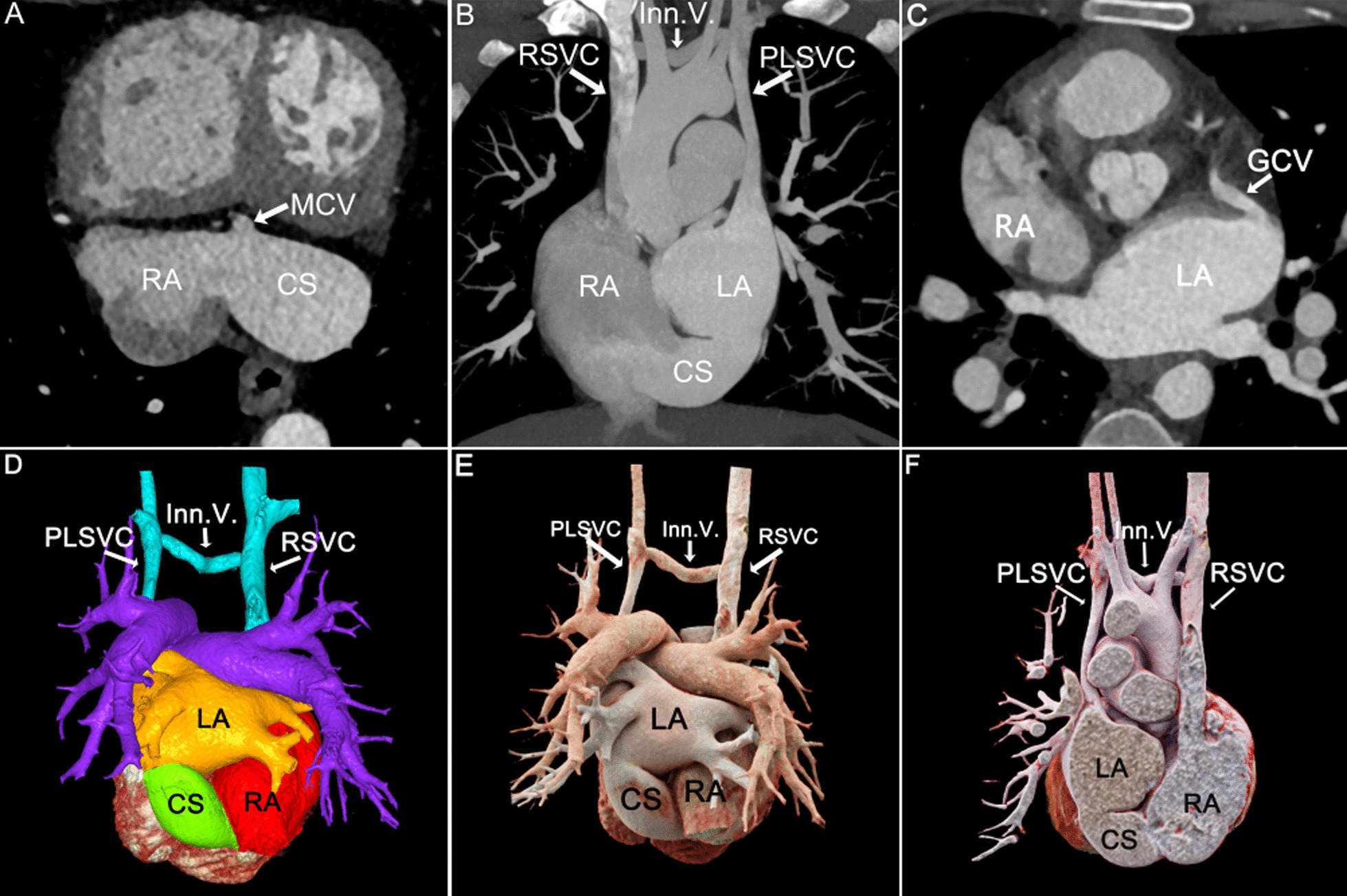
Patient No.6, type III: **A**, **B** MPR and MIP axial views showed an enlarged CS. **C** MIP coronal view showed the middle segment of the unroofed CS connecting to the LA. PLSVC converged into the LA. **D** MIP showed that variant great cardiac vein(GCV) drained into the LA. **E**, **F** VR color rendering and CR visualized the enlarged CS, PLSVC and Inn.V.. **G** CR cross-sectional view visualized the defect size of the CS. RA right atrium, CS coronary sinus, MCV middle cardiac vein, LA left atrium, RSVC right superior vena cava, PLSVC persistent left superior vena cava, Inn.V. innominate vein

**Fig. 4:**
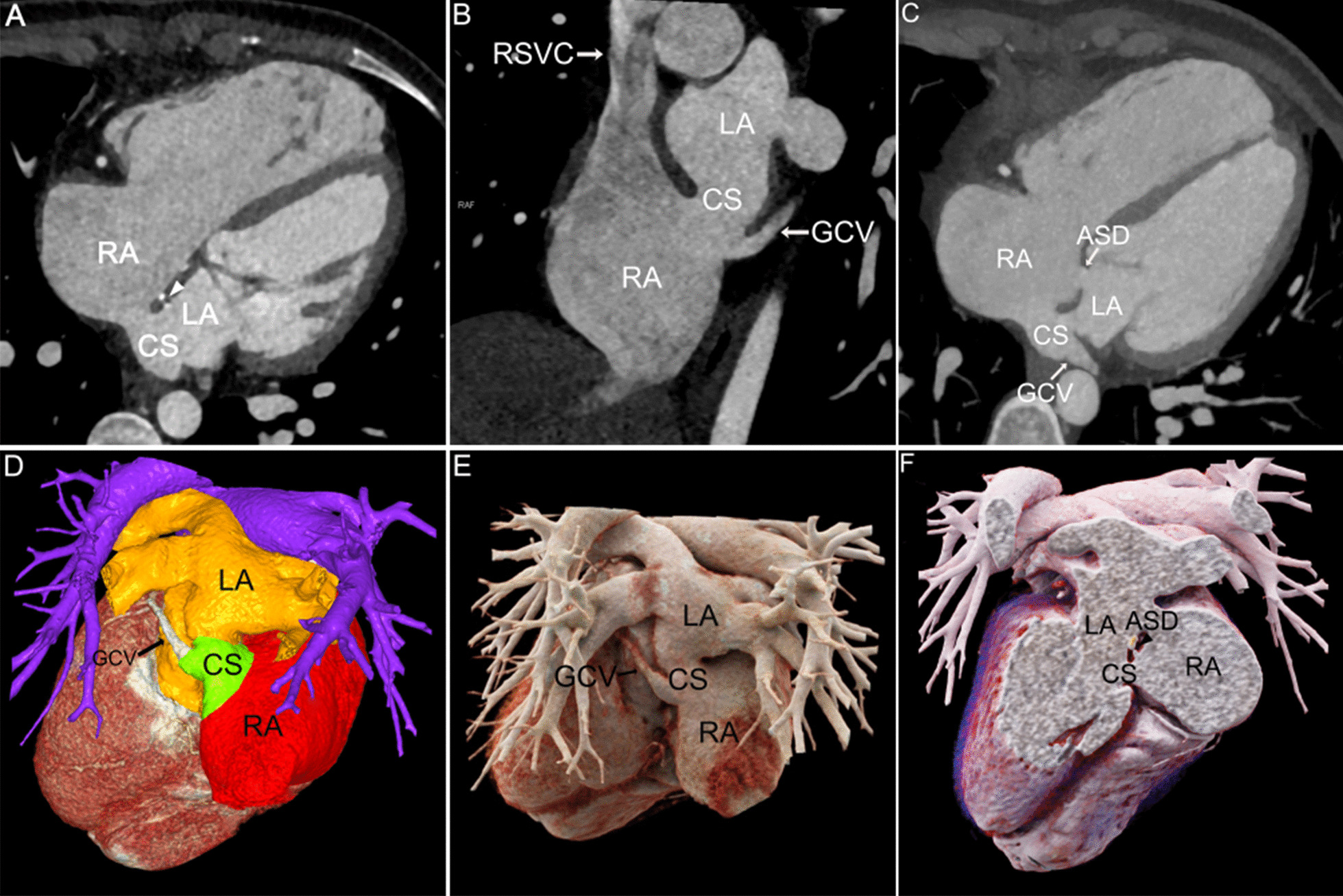
Patient No.8, type IV: **A**, **B** MPR axial and coronal views showed perforation of the parietal wall of the end segment of CS. Metal density (white arrowhead) at the atrial septum was a postoperative manifestation. **C** MIP axial view showed both the ASD and the CS wall defect. **D**, **E** VR color rendering and CR visualized the CS. **F** CR cross-sectional view provided the spatial location of the ASD and the CS defect. Postoperative metal density was visible (black arrowhead). RA right atrium, LA left atrium, CS coronary sinus, RSVC right superior vena cava, GCV great cardiac vein, ASD atrial septal defect

**Fig. 5:**
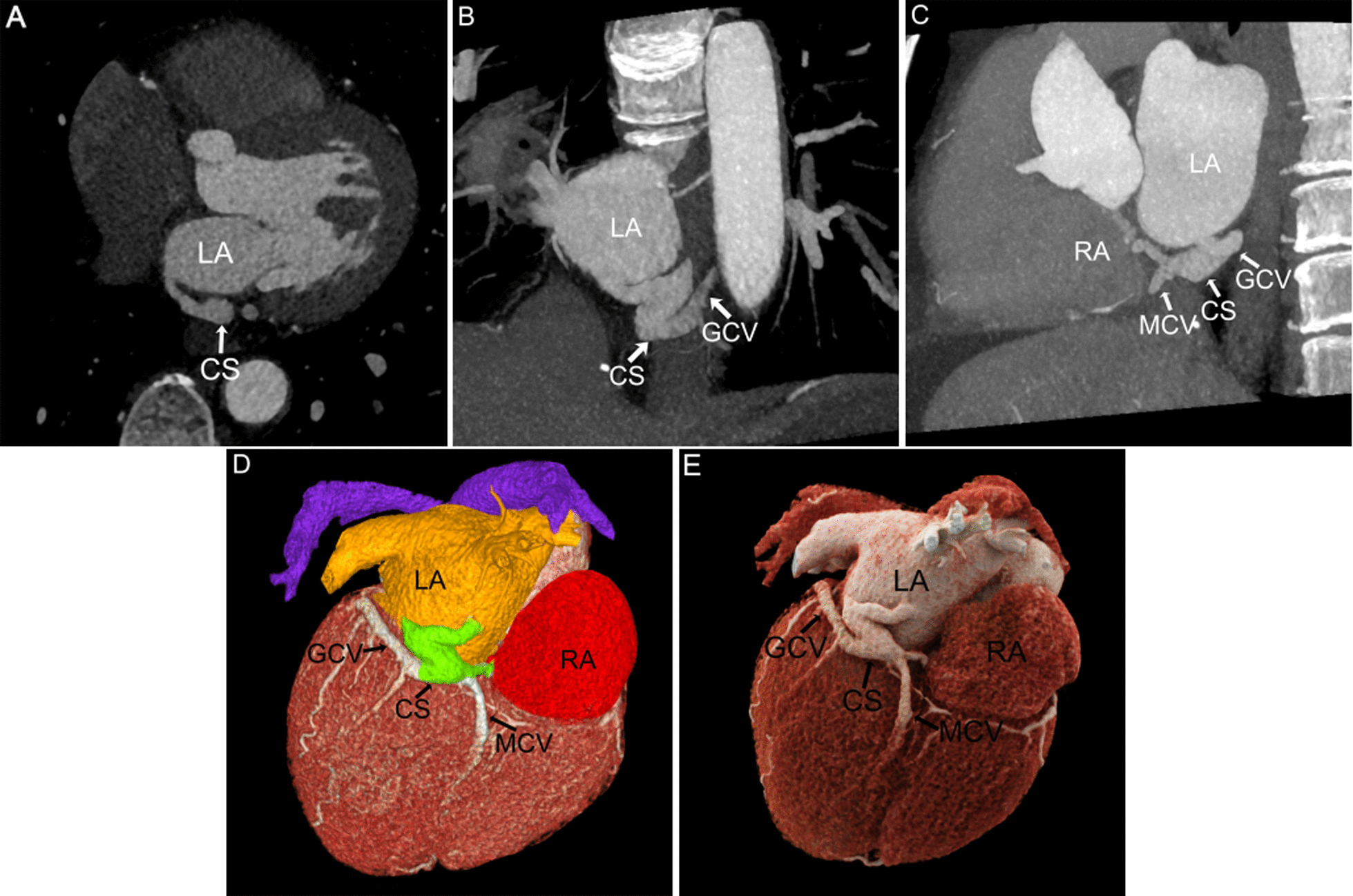
Patient No. 9, type V: **A**, **B**: MPR and MIP showed the "Z"-shaped vessel communicated the LA to the CS. **C** MIP showed the convergence of the CS into the RA. **D**, **E** VR and CR showed the spatial relationship between the CS and the right and left atrium. LA left atrium, CS coronary sinus, GCV great cardiac vein, RA right atrium, MCV middle cardiac vein

9 patients were classified into 5 groups according to Kirklin and Barratt Boyes classification of UCSS [1], including 1 case of type I (11.1%), 4 cases of type II (44.4%), 1 case of type III (11.1%), 2 cases of type IV (22.2%), and 1 case of type V (11.1%). Typical images of each type were shown in Figs. 1, 2, 3, 4 and 5.

### Comparison of TTE and CCT for the diagnosis of UCSS and other cardiovascular malformations, and the size of CS roof defect

According to the Kirklin and Barratt Boyes’s method [1], 1 case was classified as type I, 4 cases as type II, 1 case as type III, 2 cases as type IV and 1 case as type V (variant type). Only 4 of 9 CCT confirmed UCSS patients were detected by TTE (4/9, 44.4%), the sensitivity of TTE was lower compared to CCT by Fisher’s exact test (*P* < 0.05). TTE missed 1 type II (1/4, 25%), 1 type III (1/1, 100%), 2 type IV (2/2, 100%), and 1 type V (1/1, 100%). Additionally, CCT showed 12 extra malformations in these patients, only 5 of them were found by TTE (5/12, 41.7%), and TTE missed all extracardiac malformations including PLSVC, common pulmonary vein (CPV), total anomalous pulmonary vena cava (TAPVC), abnormalities in drainage of the great cardiac vein (ADG). The size of CS roof defect was from 0.74 to 6.02 cm, with an average value of 3.04 ± 1.57 cm (Table 2).

**Table 2:** Comparison of CCT and TTE for the diagnosis, and the size of CS roof defect

Patient no	Type	CCT (UCSS)	TTE (UCSS)	CCT (malformations)	TTE (malformations)	Size of CS Roof Defect (cm)
1	I	**√**	**√**	PLSVC/ASD	ASD	6.02
2	II	**√**	**√**	ASD	ASD	2.8
3	II	**√**	**√**	ASD/ct*/TAPVC/CPV	ASD/ct*	4.94
4	II	**√**	**√**	None	None	3.1
5	II	**√**	** × **	None	None	3.9
6	III	**√**	** × **	PLSVC/ADG	None	2.09
7	IV	**√**	** × **	PLSVC	None	1.72
8	IV	**√**	** × **	ASD/VSD	ASD	2.06
9	V	**√**	** × **	None	None	0.74

*CCT* cardiac CT, *TTE* transthoracic echocardiography, *ASD* atrial septal defect, *PLSVC* persistent left superior vena cava, *VSD* ventricular septal defect, ct* cor triatrium, *TAPVC* total anomalous pulmonary vena cava, *CPV* common pulmonary vein, *ADG* abnormalities in drainage of the great cardiac vein(great cardiac vein), √ UCSS was diagnosed by CCT or TTE, × UCSS wasn't diagnosed by CCT or TTE

### Surgery information

One patient in type IV with PLSVC underwent a simple atrial septal repairment before CCT. Two type II patients underwent surgery after CCT: one underwent atrial septal repairment, cor triatriatum correction, mitral and tricuspid valve replacement; the other underwent atrial septal repairment and tricuspid valvuloplasty. In these 2 cases, the CS defects were isolated to the LA by closing the CS ports, and no obvious postoperative complication was observed.

## Discussion

UCSS is a rare special type of atrial septal defect, which refers to the partial or complete defect of CS septum, causing CS to connect to LA. The clinical presentation of UCSS is not characteristic, and when it is combined with other cyanotic cardiac malformations, an accurate early diagnosis is even more difficult [11]. Therefore, the preoperative diagnosis of UCSS relies heavily on imaging, such as TTE, CCT, MRI, etc.

With high temporal resolution and advantages of 3D visualization of cardiac anatomy, CCT technology has gradually become the preferred modality for cardiovascular disease in most medical centers [12, 13]. CCT can reconstruct the image in any plane without losing spatial resolution, and minimize the partial volume effect [14]. MPR images based on the short axis of the heart are called "CS views", which show the entire course of CS (Figs. 1A, 2, 3, 4, 5A) [15], while reconstructions based on the long axis of the heart are more conducive to display the CS defect and are used to diagnose and classify UCSS clearly (Figs. 1B, 2D, 4B). MIP can provide a good "roadmap" to vascularity (Figs. 1C, 3C, 5B, 5C) [16]. The interactive evaluation of MPR images and MIP images can better understand the course of CS, PLSVC and their adjacent tissue. VR can provide density and spatial information, and be customized in real time to display anatomical details from different perspectives [17]. For the display of UCSS, VR is not only able to present the entire spatial relationship of RSVC-RA-CS-LA-PLSVC-Inn.v. (Figs. 1D, 3D) in its entirety, but also to reproduce complex vascular connections in 3D, especially for UCSS type V, VR can directly diagnose and classify it (Fig. 5D). CR technology is a new postprocessing technology. Compared with VR, CR technology can obtain highly realistic 3D images with photo level authenticity, further enhance the evaluation of spatial relationship and improve the perception of depth and shape [18]. Tomographic imaging of CR can isolate or remove specific anatomical features such as coronary trees, calcification and bone, as well as lungs and airways. Clip planes and crop boxes can be applied to cut the rendered volume [19]. The application of CR technology allows for a near-realistic view of the spatial relationship between CS defects and combined malformations (Figs. 1F, 2G, 3F, 4F). In this study, CCT applied multiple postprocessing methods as described above to clearly diagnose all 9 patients and further typed the UCSS. In addition, our patients also underwent TTE, but only 4 UCSS patients (44.4%) were detected, which was roughly consistent with the results of *Raghib, G* (46%) [20]. And it is worth noting that TTE missed types III-V patients completely. The reason may be that compared with types I-II, types III-V have smaller CS defects, which are difficult to show directly with the limited acoustic window. Thus, in the UCSS diagnosis, TTE still has a non-negligible disadvantage compared to CCT.

UCSS is often associated with other malformations, such as atrial septal defect, ventricular septal defect, uniatrial heart, completely abnormal pulmonary venous drainage and tetralogy of Fallot [21]. The excellent temporal (millisecond) and spatial (sub-millimetre) resolution of the CCT undoubtedly ensures the accuracy of the diagnosis of cardiac malformations and the description of the overall intracardiac-vascular-thoracic structures [22, 23]. In this cohort, 12 extra malformations were diagnosed by CCT comparied to 5 diagnosed by TTE (41.7%). TTE showed only ASD and cor triatrium but neglected PLSVC, pulmonary vein structural anomalies, which certainly demonstrated the superiority of CCT for the diagnosis of extracardiac malformations in UCSS. Moreover, CCT identified 2 rare cases of UCSS. The one was a type III patient with combined ectopic drainage of the great cardiac vein into the left atrium (Fig. 3D), according to literature review, this is the first report in patients with UCSS. Previously, only 4 cases of simple ectopic drainage of left atrial great cardiac vein were reported [10, 24]. The other type II patient was accompanied by cor triatriatum and total anomalous pulmonary vena cava (TAPVC) (Fig. 2), the possibly similar case was also reported by *Kwak, J* [25].

The surgical option for UCSS depends on the presence of PLSVC and the presence of a vascular bridge between RSVC and PLSVC [26]. When not combined with PLSVC, it is enough to use a patch to repair the gap between CS and LA, or ligate coronary sinus ostium of RA [27]. Preoperative observation of the CS defect size and morphology by CCT to assess the ease of patch placement is important. In this study, with CCT suggestions, 2 type II patients rescheduled the surgical strategy from patch repairment to close coronary sinus ostium, due to large CS defects were prone to loosening. If PLSVC is combined, central venous pressure (CVP) needs to be measured to determine if the RSVC and LSVC are obstructed or connected. And if there is no Inn.V., the LSVC should not be ligated [28]. The CCT reproductive coronal views allow visual assessment of PLSVC course, diameter, and the presence or absence of the Inn.V., which can assist in surgical planning and postoperative follow-up.

## Limitations

This was a retrospective study aimed at providing mainly descriptive images to evaluate the CCT advantages in diagnosing UCSS and its malformations. However, small sample size limited the statistical power of analysis. In addition, UCSS patients should be followed up longer to understand the prognosis and treatment results.

## Conclusions

UCSS is a rare congenital heart disease, and its clinical presentation lacks diagnostic specificity. CCT has a advantage in the diagnosis of UCSS, differentiating subtypes of UCSS, depicting the size of the coronary sinus defect, describing other associated cardiovascular anomalies. The varied presentation of UCSS with the CCT postprocessing techniques allows clinicians to have a more direct and comprehensive view of the lesion, which can improve clinician choice of treatment mode or surgical plan and the assessment of prognosis.

## Data Availability

All data generated or analysed during this study are included in this published article. The data are available from the corresponding author on reasonable request.
